# Supplementary material for: Control of SARS-CoV-2 infection by MT1-MMP-mediated shedding of ACE2
Source: Nat Commun. 2022 Dec 23;13:7907. doi: 10.1038/s41467-022-35590-x (PMC9780620; doi:10.1038/s41467-022-35590-x)

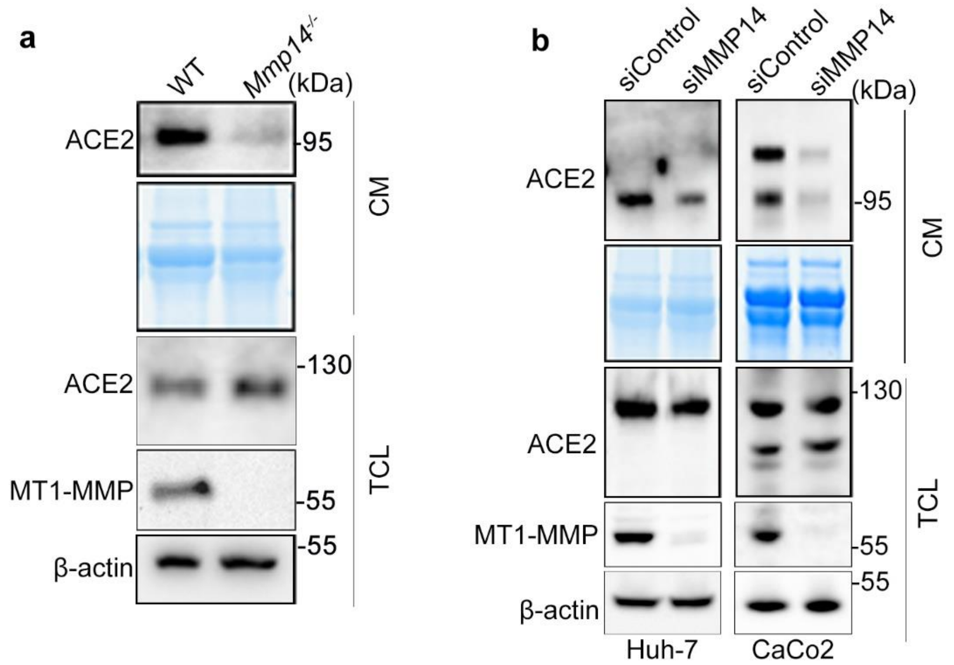

Supplementary Figure 1. MT1-MMP deficiency suppresses the release of sACE2 in kidney epithelial cells

(a) Western blotting analyses on the expression of ACE2 in wild-type (WT) and *Mmp14*$^{-/-}$ mouse primary kidney epithelial cells. The level of soluble ACE2 (solACE2) was detected in conditioned media. Coomassie stained membrane was a loading control for conditioned medium. (n=3) **(b)** Western blotting analyses on the levels of solACE2 and memACE2 in Huh-7 and CaCo2 cells transfected with either control siRNA or siMMP14. (n=3)

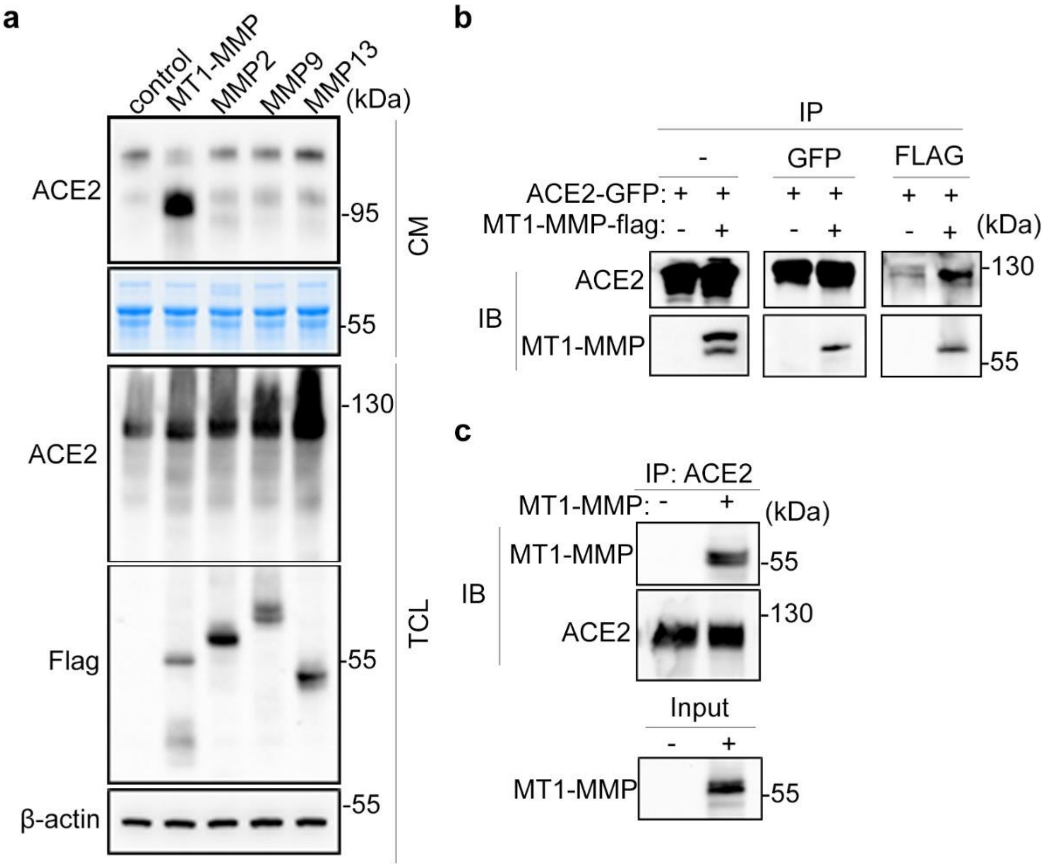

Supplementary Figure 2. Physical interaction between MT1-MMP and ACE2

(a) Western blotting analyses on the levels of memACE2 in the total cell lysate (TCL) and solACE2 in the conditioned media (CM) derived from ACE2-expressing HEK293T cells transfected with plasmids expressing different MMPs including MT1-MMP, MMP2, MMP9 and MMP13. Coomassie stained membrane served as a loading control for conditioned media. (n=3) **(b)** HEK-293T cells were cotransfected with ACE2-GFP and MT1-MMP-FLAG plasmids. Cell lysates were subjected to immunoprecipitation with the indicated antibodies, followed by western blotting analyses. (n=3) **(c)** Recombinant ACE2 protein was incubated with MT1-MMP derived from the lysate of HEK293T cells transfected with plasmid expressing MT1-MMP, followed by the immunoprecipitation with the anti-ACE2. The expression of MT1-MMP was detected in the ACE2 immunoprecipitate by western blotting. (n=3)

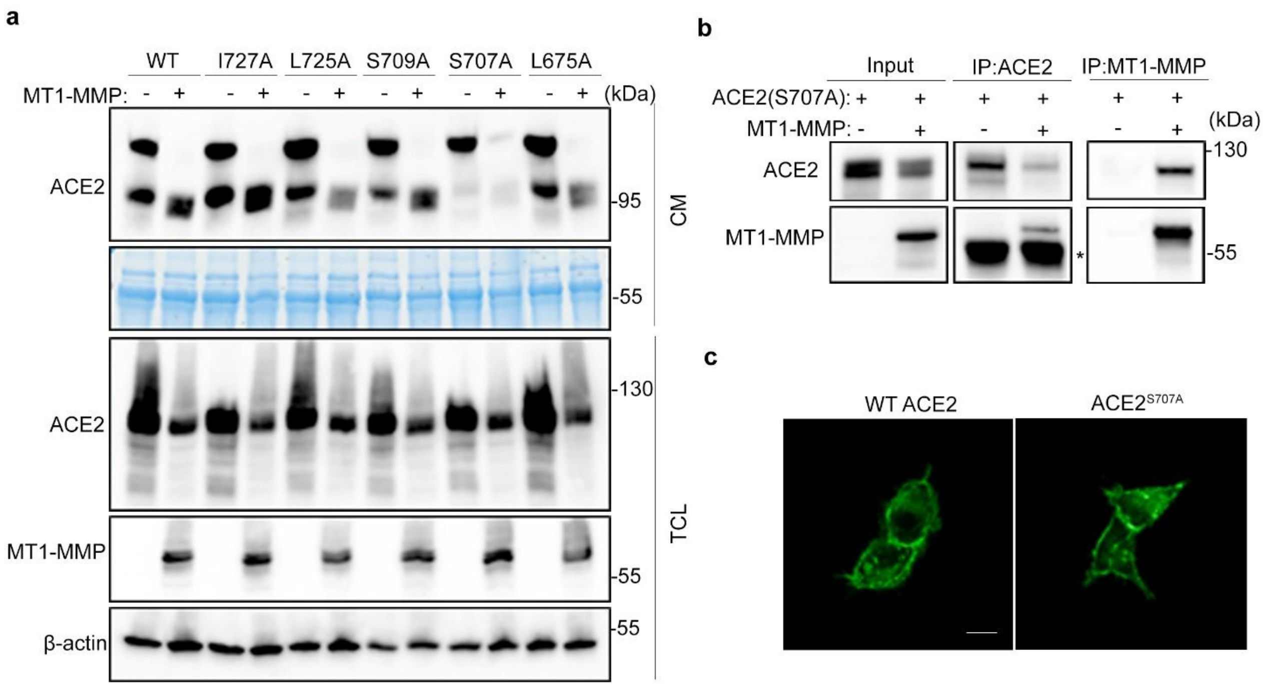

Supplementary Figure 3. The identification of MT1-MMP cleavage site of ACE2

(a) S707A mutation abolished the ACE2 shedding by MT1-MMP. 293T cells were co-transfected with MT1-MMP and different ACE2 mutants including wild-type ACE2, $ACE2^{I727A}$, $ACE2^{L725A}$, $ACE2^{S709A}$, $ACE2^{S707A}$ and $ACE2^{L675A}$. The levels of memACE2 in total cell lysate (TCL) and solACE2 in conditioned media (CM) were detected by western blotting. Coomassie stained membrane served as a loading control for conditioned media. (n=3) **(b)** The physical interaction between MT1-MMP and $ACE2^{S707A}$ was examined by co-immunoprecipitation using the cell lysates derived from HEK293T cells co-expressing MT1-MMP and $ACE2^{S707A}$. (n=3) **(c)** Immunofluorescent staining of ACE2 in HEK293T cells with ectopic expression of either wild-type ACE2 or $ACE2^{S707A}$. (n=3) (scale bars: 20μm)

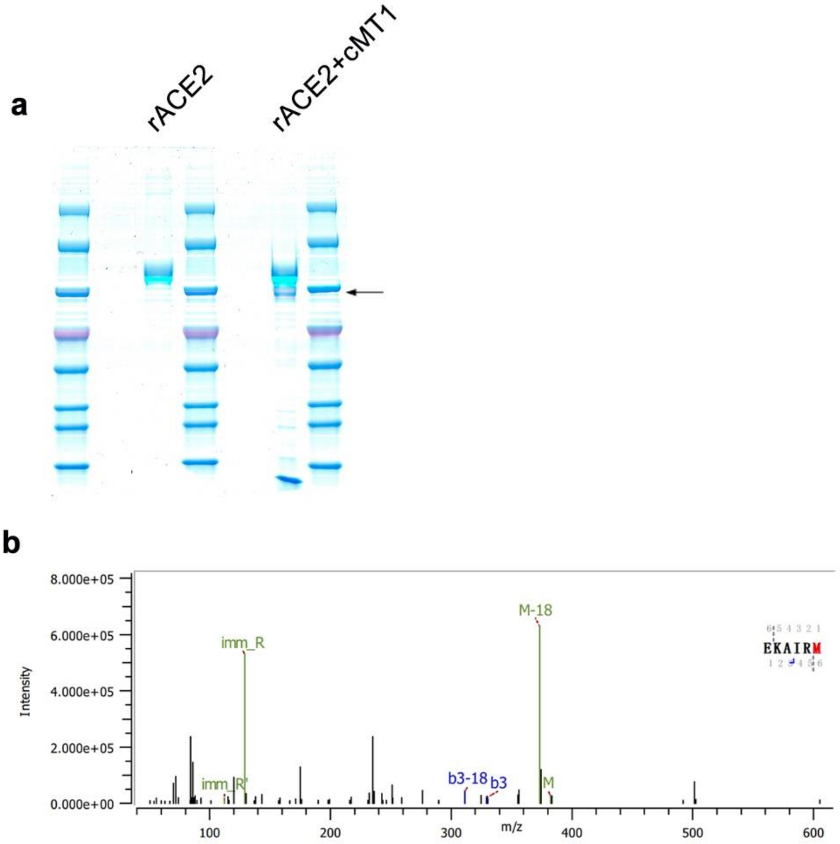

Supplementary Figure 4. Mass-spectrometry analyses of ACE2 fragment

(a) Recombinant ACE2 was cleaved by recombinant catalytic domain of MT1-MMP (cMT1) in the in vitro cleavage assay, following by SDS-PAGE. The protein bands in the gel were visualized by Coomassie blue staining. The ACE2 truncated fragment generated by MT1-MMP cleavage, indicated by the black arrow, was sequenced by mass spectrometry (MS) analyses followed by tandem MS/MS as shown in **(b)**. (n=3)

ACE2(691-710)

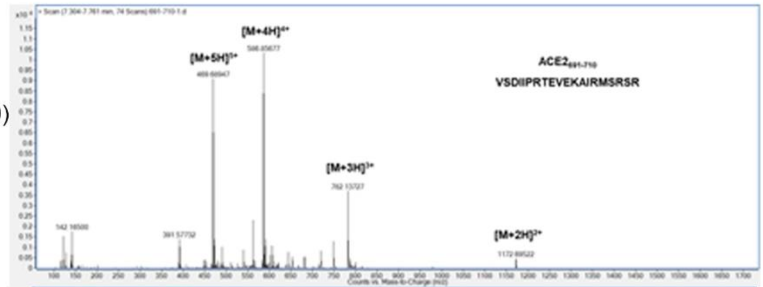

ACE2(691-710) + cMT1

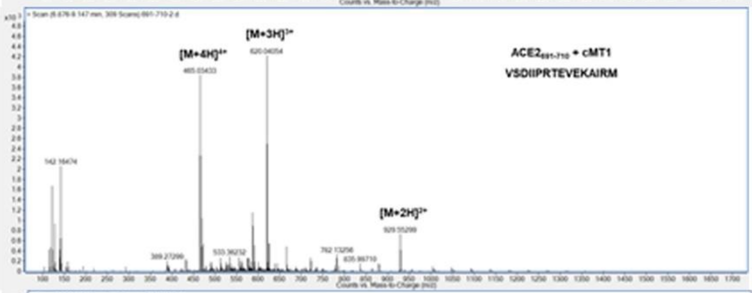

ACE2(691-710) +cMT1+ EDTA

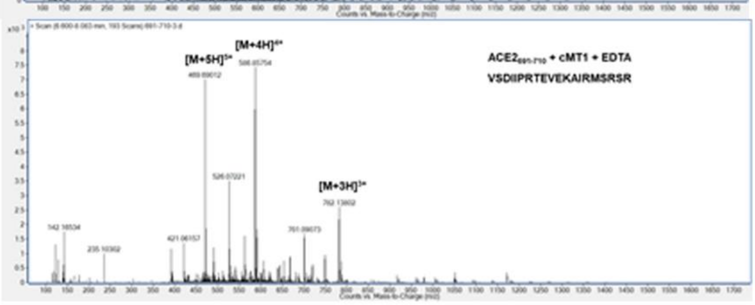

ACE2(701-720)

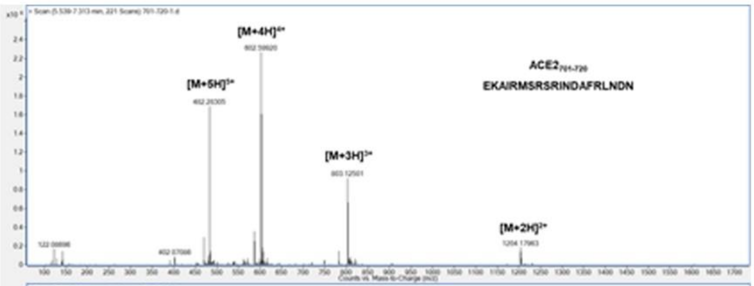

ACE2(701-720) + cMT1

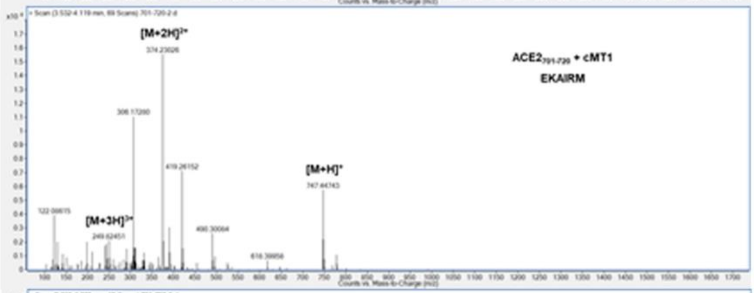

ACE2(701-720) +cMT1

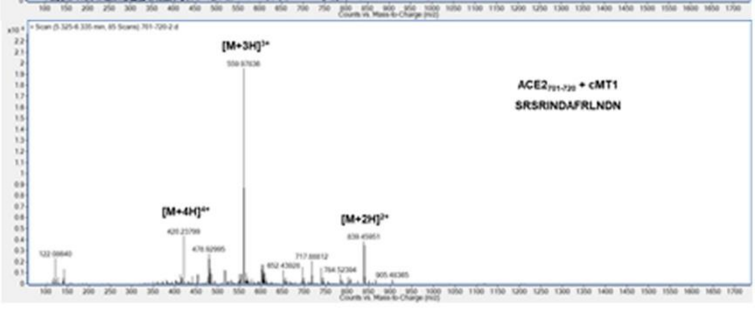

Supplementary Figure 5. Identification of MT1-MMP cleavage site of ACE2 by mass-spectrometry

Mass spectrometry analysis of ACE2 peptides 691^{V}-710^{R} and 701^{E}-720^{N} after incubation with the catalytic domain of MT1-MMP. Control experiments were performed in the presence of EDTA. In the presence of recombinant MT1-MMP, the intact peptide 691^{V}-710^{R} was cleaved into a smaller fragment that was identified as VSDIIPRTEVEKAIRM by tandem MS/MS. Consistently, the intact peptide 701^{E}-720^{N} was digested into two smaller fragments that were identified as EKAIRM and SRSRINDAFGLNDN respectively. MT1-MMP cleaved both peptides at M706-S.

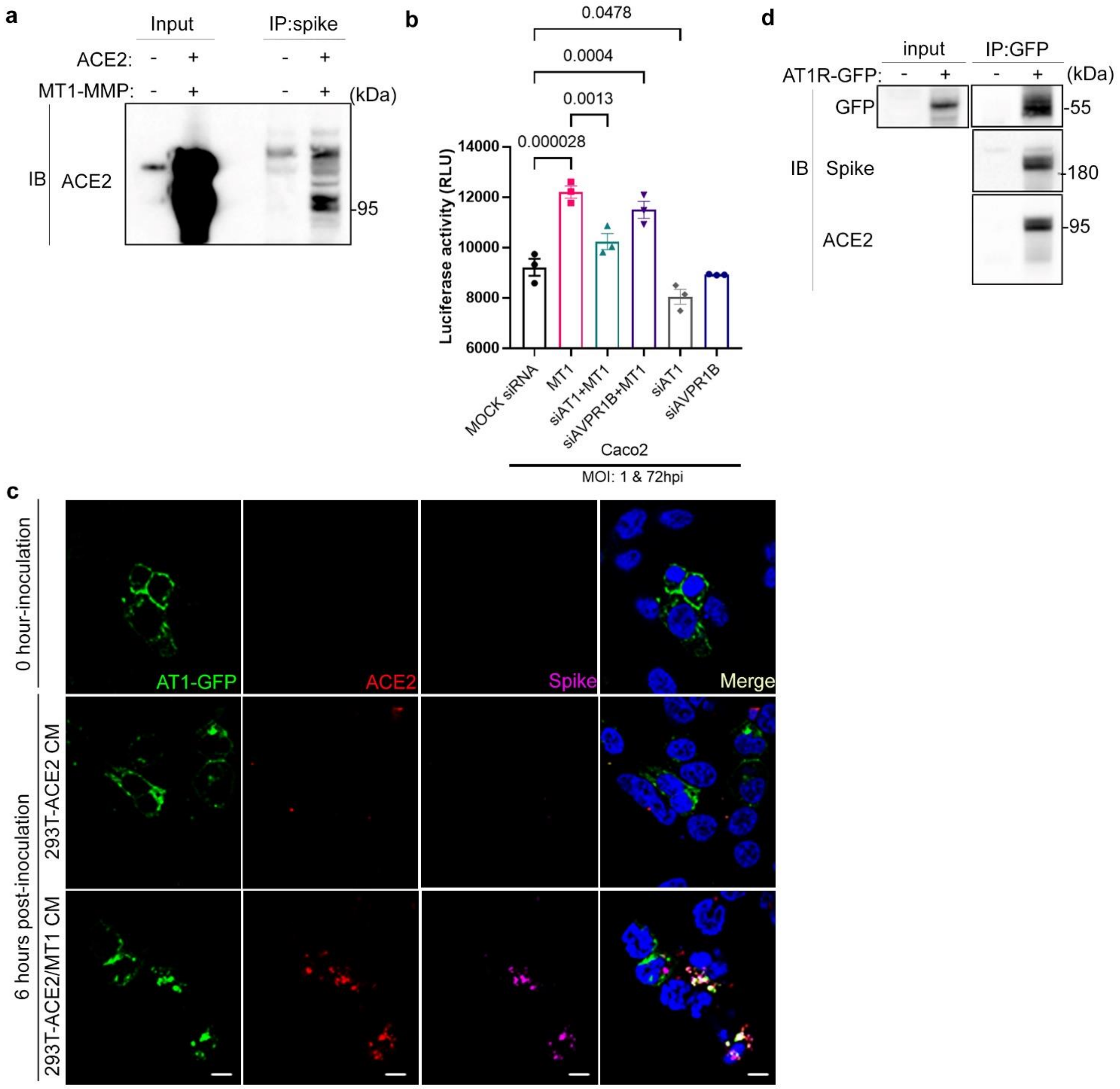

Supplementary Figure 6. sACE2 released by MT1-MMP mediates cell entry of SARS-CoV-2 through AT1R

(a) Conditioned media derived from HEK-293T cells co-transfected with/without ACE2 and MT1-MMP were incubated with recombinant spike proteins of SARS-CoV-2. Subsequently, the protein complex was immunoprecipitated with spike-specific antibodies and were analyzed by western blotting using specific antibodies. (n=3) **(b)** Caco2 cells were firstly transduced with adenoviral vector of MT1-MMP and then transfected with siRNAs targeting either AT1 or AVPR1B, followed by the infection with SARS-CoV-2 pseudotyped virus. Luciferase levels were detected at 72 h post-infection. (n=3 biologically independent experiments) **(c)** Immunofluorescent staining of SARS-CoV-2 spike protein, solACE2 and AT1-transfected 293T cells. Non-permissive HEK293T cells were firstly

transfected with GFP-tagged AT1 and then inoculated with recombinant SARS-CoV-2 spike protein along with conditioned media derived from 293T cells expressing ACE2 only or ACE2 with MT1-MMP. The cells were immunostained with anti-S (purple) and anti-ACE2 antibodies (red), and counterstained with DAPI to label nuclei (blue). (scale bars: 20μm) (n=3 biologically independent experiments) **(d)** HEK293T cells were firstly transfected with AT1R-GFP and then inoculated with the HEK293T conditioned media containing recombinant SARS-CoV-2 spike proteins and solACE2 released by MT1-MMP. The protein complex of AT1R-GFP was purified by immunoprecipitation, followed by western blotting analyses on the expression of spike proteins and ACE2. (n=3) The data are shown as the means ± SEM from three independent experiments. P values were calculated using one-way ANOVA for **(b)**. Source data are provided as a Source Data file.

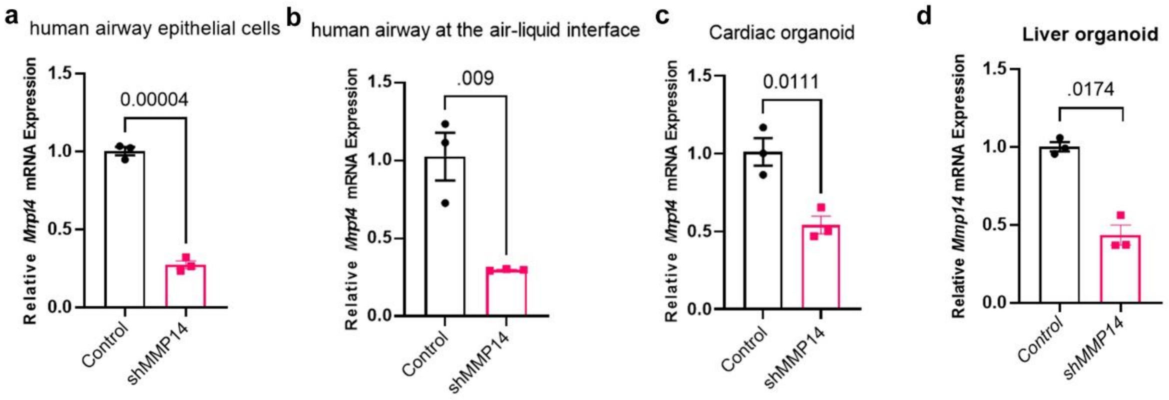

Supplementary Figure 7. Validation of MT1-MMP knockdown in primary culture

(a-d) qPCR analyses on the mRNA expression of MT1-MMP in human bronchial epithelial cells **(a)** (n=3 biological independent experiments), primary human bronchial epithelial 3D cultures with an air-liquid interface (ALI) **(b)** (n=3 biological independent experiments), human cardiac organoids formed by iPSC-derived cardiomyocytes **(c)** (n=3 biological independent experiments) and human liver organoids **(d)** (n=3 biological independent experiments) after lentiviral transduction with shRNA or shMMP14. The data are shown as the means ± SEM. unpaired two-tailed *t*-test for (**a-d)** Source data are provided as a Source Data file.

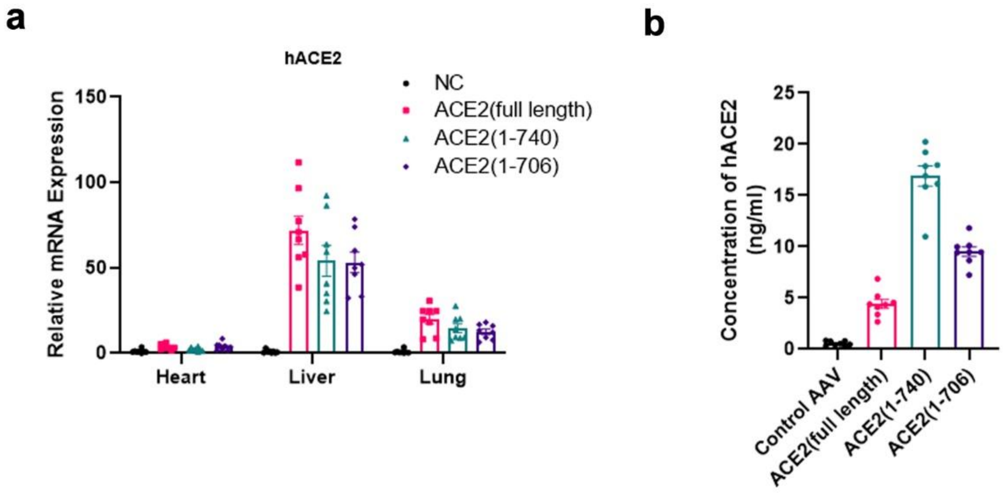

Supplementary Figure 8. Tissue distribution of AAV transduction in mice

(a) qPCR analyses of the expression of human ACE2 in mice expressing solACE2$_{1\text{-}706}$/ solACE2$_{1\text{-}740}$/ACE2 (full length). (n=7 for AAV-control; n=7 for AAV-hACE2(1-706); n=6 for AAV-hACE2 (1-740)). **(b)** The level of human ACE2 in the plasma from mice expressing solACE2$_{1\text{-}706}$/ solACE2$_{1\text{-}740}$ was measured by ELISA assay (n=7 for AAV-control; n=7 for AAV-hACE2(1-706); n=6 for AAV-hACE2 (1-740)). The data are shown as the means ± SEM. Source data are provided as a Source Data file.

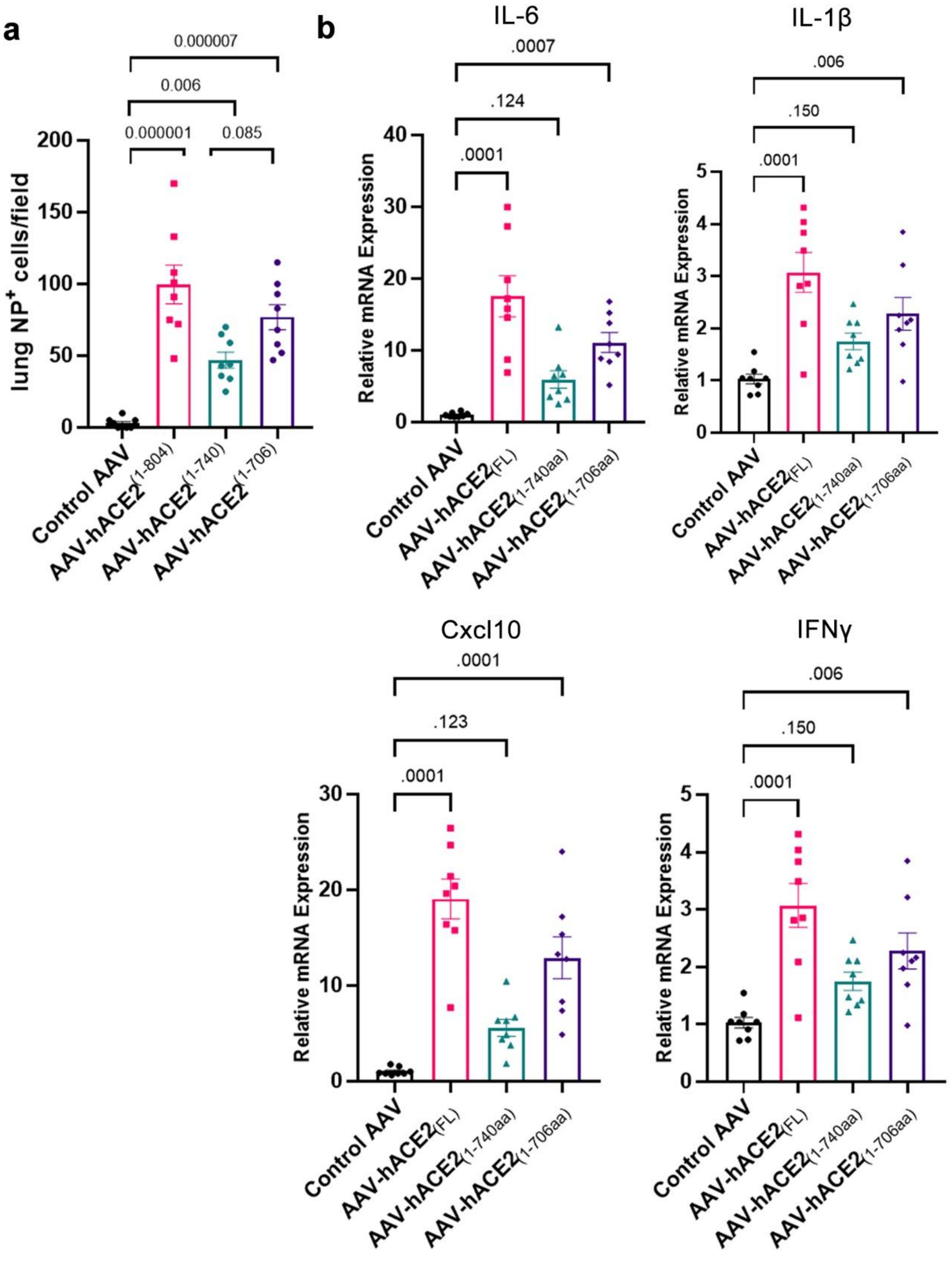

Supplementary Figure 9. solACE2 facilitates SARS-CoV-2 infection in non-permissive laboratory mice

(a) The number of viral antigen-presenting cells in lung sections of mice (50× field) expressing solACE2$_{1-706}$/ solACE2$_{1-740}$/ACE2$_{\text{full length (FL)}}$ (n=8 for AAV-control; n=8 for AAV-hACE2(1-706); n=8 for AAV-hACE2 (1-740); n=8 for AAV-hACE2(full length)). **(b)** qPCR analyses of inflammatory cytokines including IL-6, IL-1β, Cxcl10 and IFN-γ were performed in lung tissues of mice expressing

solACE2$_{1\text{-}706}$/ solACE2$_{1\text{-}740}$/ACE2$_{\text{full length (FL)}}$ (n=8 for AAV-control; n=8 for AAV-hACE2(1-706); n=8 for AAV-hACE2 (1-740); n=8 for AAV-hACE2(full length)). The data are shown as the means ± SEM. One-way ANOVA for **a-b.** Source data are provided as a Source Data file.

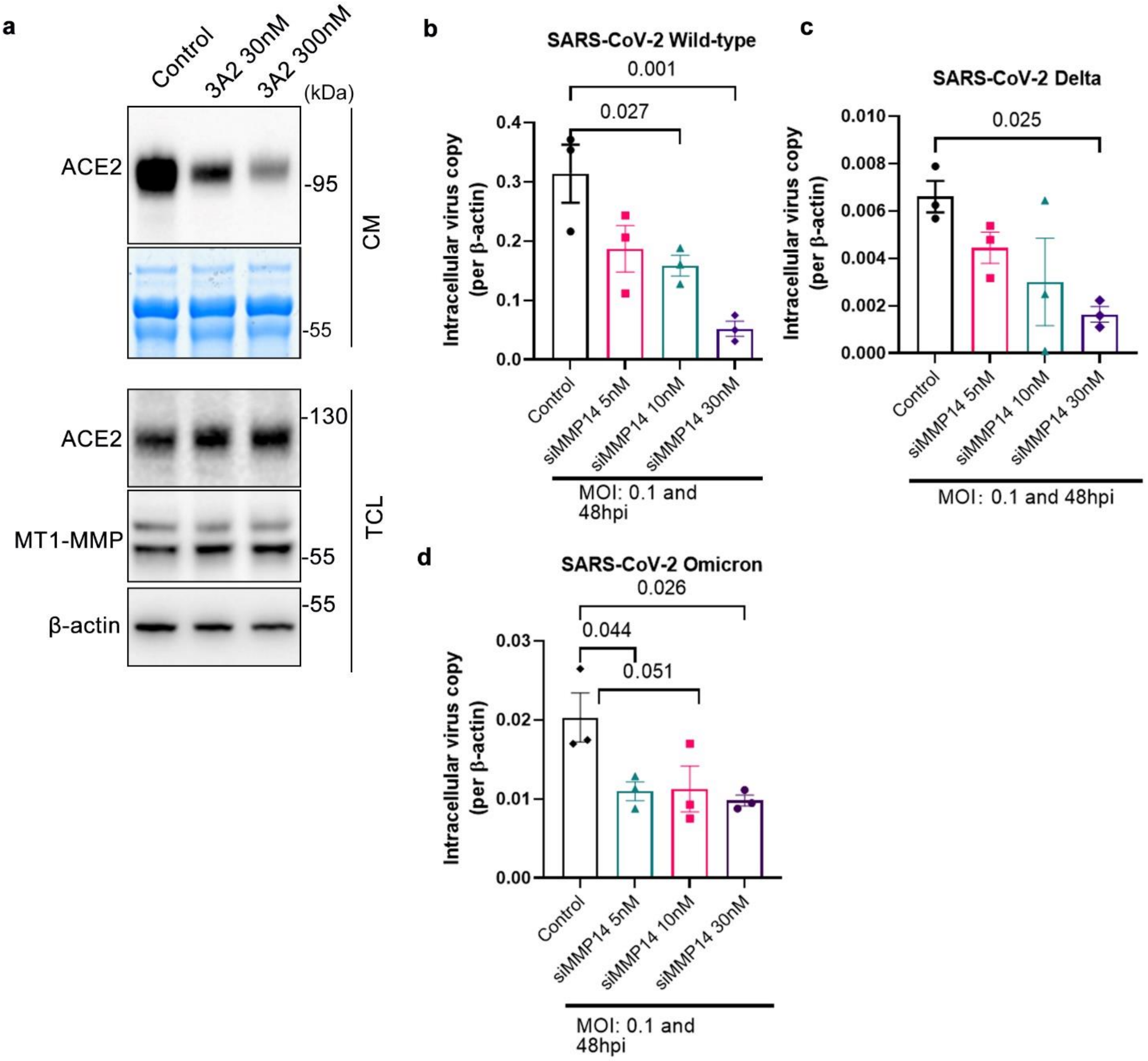

Supplementary Figure 10. MT1-MMP knockdown suppresses solACE release and the infection of SARS-CoV-2 variants of concern

(a) Primary human bronchial epithelial 3D cultures with an air-liquid interface (ALI) pre-treated with different concentrations of 3A2 were infected with SARS-CoV-2 wild-type strain. The levels of memACE2 in total cell lysate (TCL) and solACE2 in conditioned media (CM) were examined by western blotting. Coomassie stained membrane served as a loading control for conditioned media. (n=3) **(b-d)** CaCo2 cells were transfected with 5nM, 10nM or 30nM siMMP14 or control siRNA, followed by infection with SARS-CoV-2 wild-type strain **(b)**, SARS-CoV-2 delta variants **(c)** or SARS-CoV-2 omicron variants **(d)** (MOI=0.1) for 48 h. Viral infection were assayed by RT-qPCR for SARS-CoV-2 progeny titers. The data are shown as the means ± SEM from three independent experiments. P values were calculated using one-way ANOVA. Source data are provided as a Source Data file.

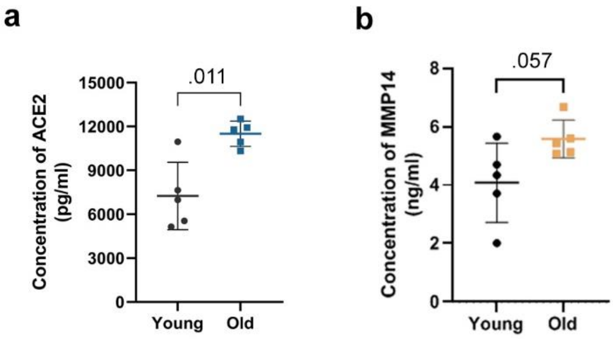

Supplementary Figure 11. The upregulation of ACE2 and MT1-MMP in aged mice

Plasma levels of ACE2 **(a)** and MMP14 **(b)** in young and old mice were measured by ELISA. The data are shown as the means ± SEM. **p < 0.01, unpaired two-tailed *t*-test. (n=5). Source data are provided as a Source Data file.

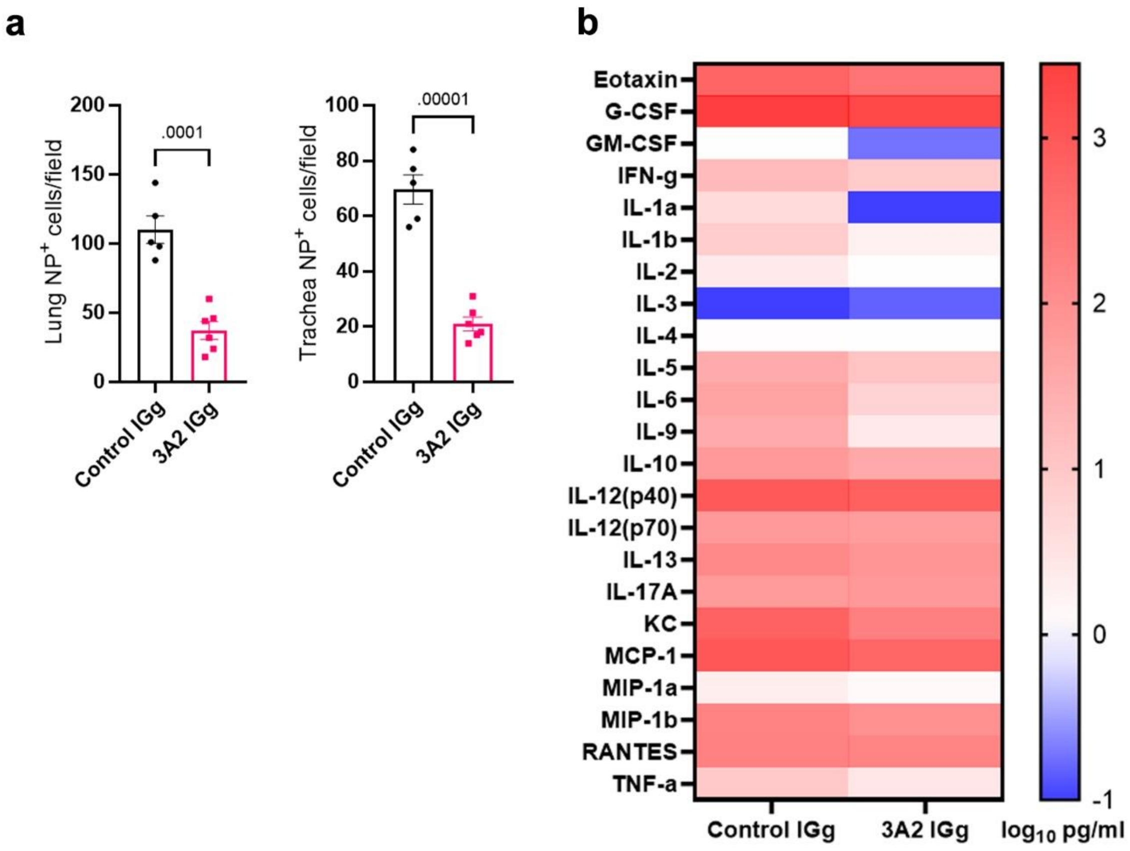

Supplementary Figure 12. 3A2 treatment protects aged mice from SARS-CoV-2 infection

(**a**) The number of viral antigen-presenting cells in lung and tracheal sections of 3A2/control IgG-treated mice challenged with mouse-adapted SARS-CoV-2 (n=5 for IgG control; n=6 for 3A2) (50× field) **(b)** Microarray analyses of inflammatory cytokines and chemokines in lung tissues from 3A2/control IgG-treated mice challenged with mouse-adapted SARS-CoV-2 (n=5 for IgG control; n=6 for 3A2)The data are shown as the means ± SEM. Unpaired two-tailed t-test for **(a).** Source data are provided as a Source Data file.

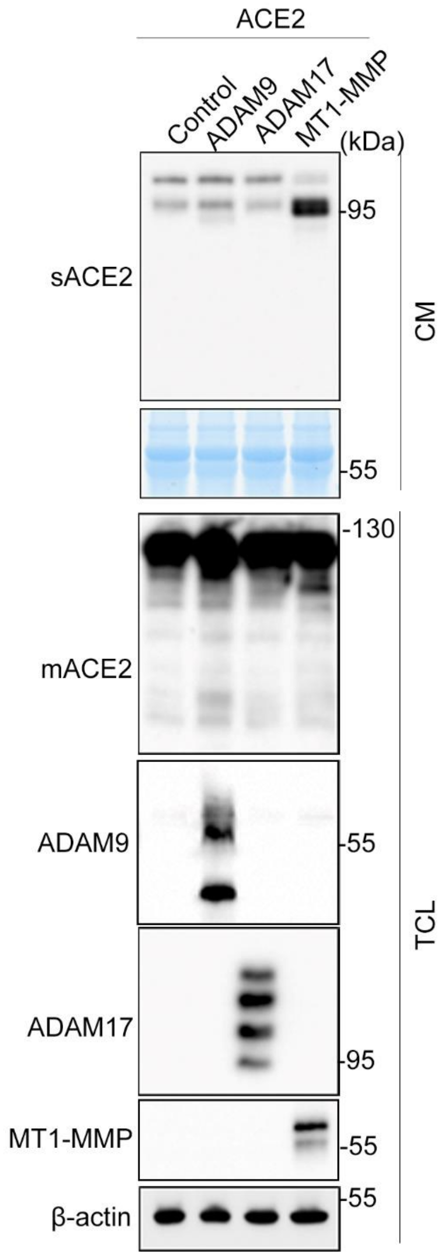

Supplementary Figure 13. Comparison of ACE2 shedding by various proteases

HEK293T cells were co-transfected with ACE2 and either control, ADAM9, ADAM17 or MT1-MMP plasmids. The expression of ACE2 was examined in conditioned media and total cell lysates by western blotting using specific antibodies. Coomassie stained membrane served as a loading control for conditioned medium. (n=3)

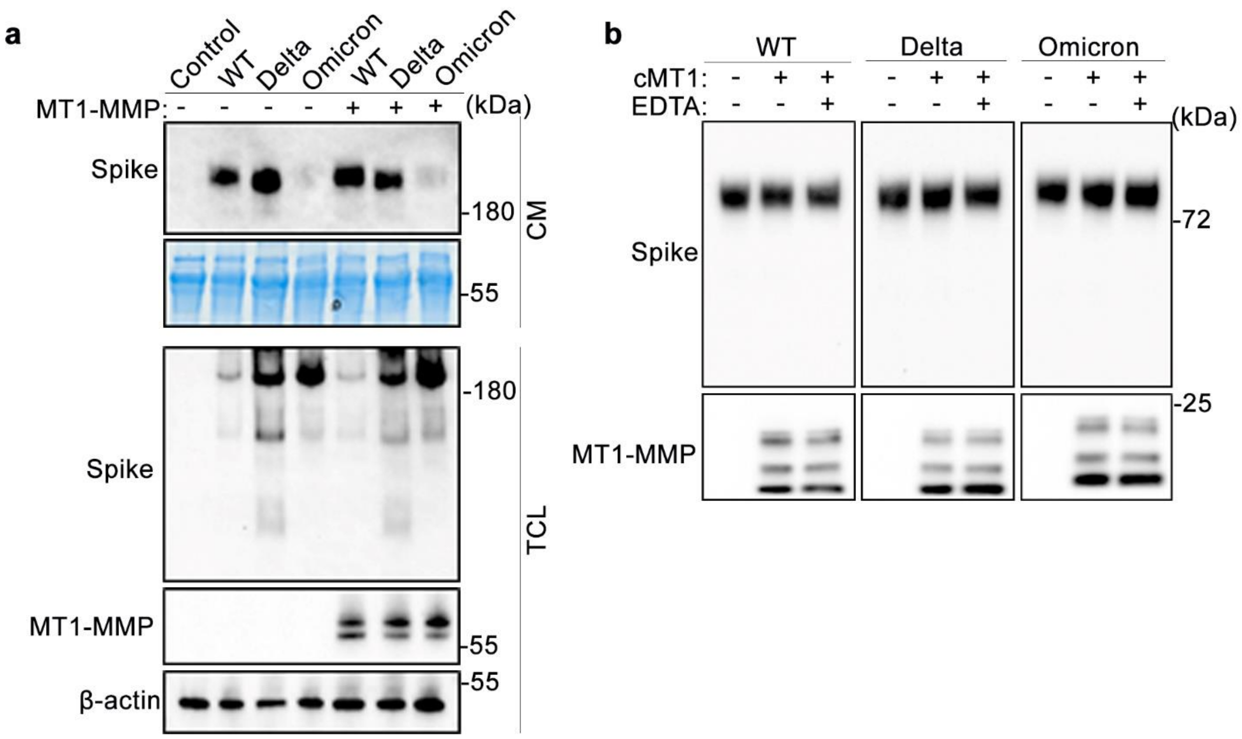

Supplementary Figure 14. MT1-MMP does not process SARS-CoV-2 spike proteins

(a) HEK293T cells were transfected with plasmids expressing MT1-MMP and SARS-CoV-2 Spike derived from wild-type, delta and omicron variants, followed by western blotting analyses of spike expression. (n=3) **(b)** Recombinant SARS-CoV-2 Spike proteins derived from wild-type, delta and omicron variants were incubated with the catalytic domain of MT1-MMP (cMT1) *in vitro,* followed by western blotting analyses of spike levels. (n=3)

Supplementary Table 1 The primers for the ACE2 mutations were (5'-3'):

Mutation Sites	Forward	Reverse
L675A	GTGCGAGTGGCTAATGCGAAACCAAGAATCTCC	GGAGATTCTTGGTTTCGCATTAGCCACTCGCAC
S707A	AAGGCCATCAGGATGGCCCGGAGCCGTATCAAT	ATTGATACGGCTCCGGGCCATCCTGATGGCCTT
S709A	ATCAGGATGTCCCGGGCCCGTATCAATGATGCT	AGCATCATTGATACGGGCCCGGGACATCCTGAT
L725A	AACAGCCTAGAGTTTGCGGGGATACAGCCAACA	TGTTGGCTGTATCCCCGCAAACTCTAGGCTGTT
I727A	CTAGAGTTTCTGGGGGCACAGCCAACACTTGGA	TCCAAGTGTTGGCTGTGCCCCCAGAAACTCTAG

Figure 1

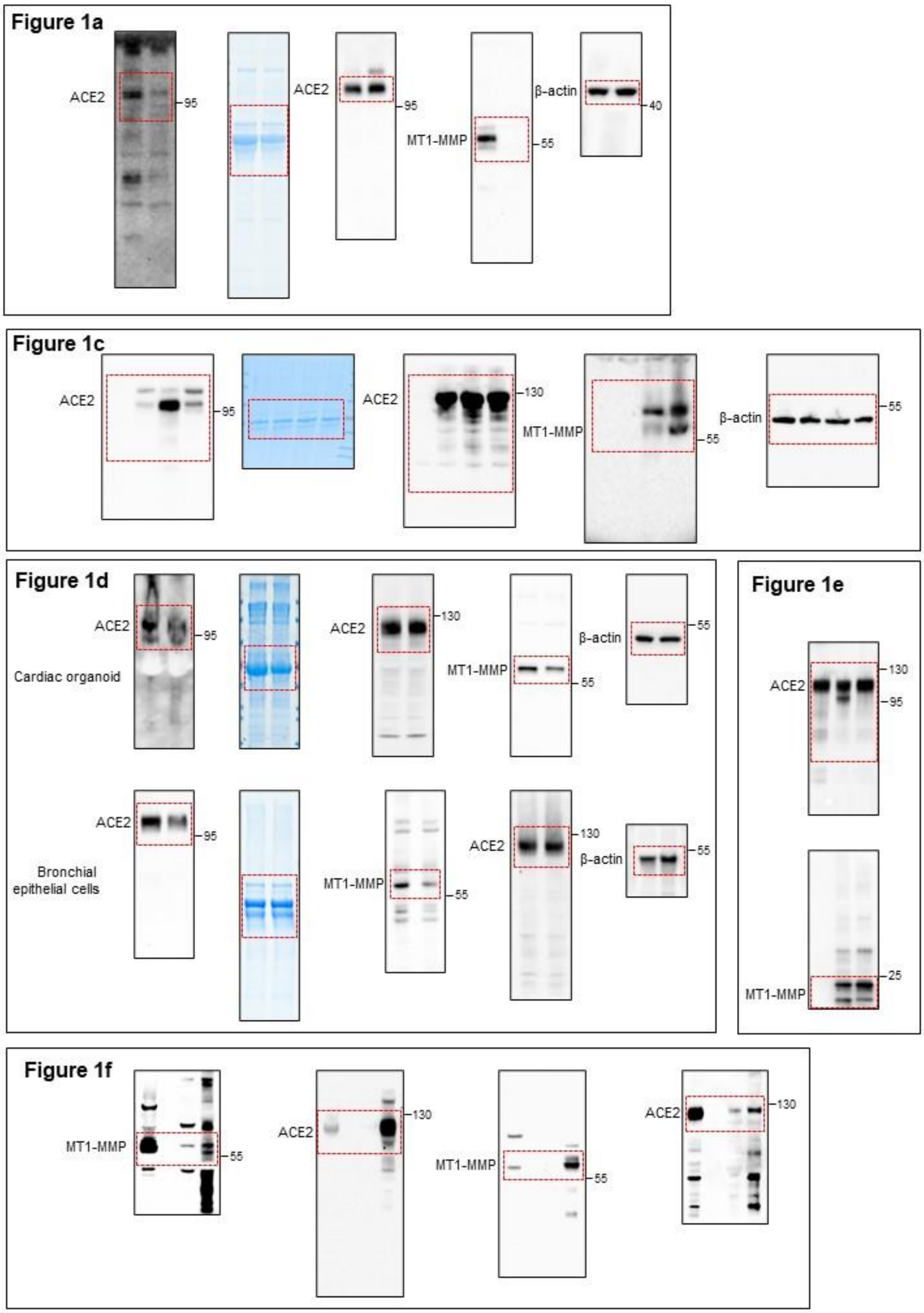

Figure 1a
ACE2
95
ACE2
95
MT1-MMP
55
β-actin
40
Figure 1c
ACE2
95
ACE2
130
MT1-MMP
55
β-actin
55
Figure 1d
ACE2
95
Cardiac organoid
ACE2
130
MT1-MMP
55
β-actin
55
ACE2
95
Bronchial epithelial cells
MT1-MMP
55
ACE2
130
β-actin
55
Figure 1e
ACE2
130
95
25
MT1-MMP
Figure 1f
MT1-MMP
55
ACE2
130
MT1-MMP
55
ACE2
130

Figure 3

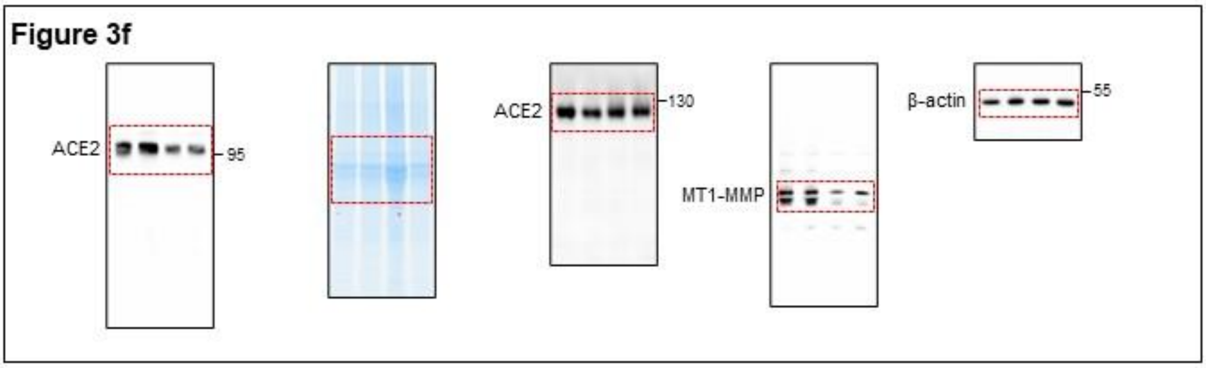

Figure 7

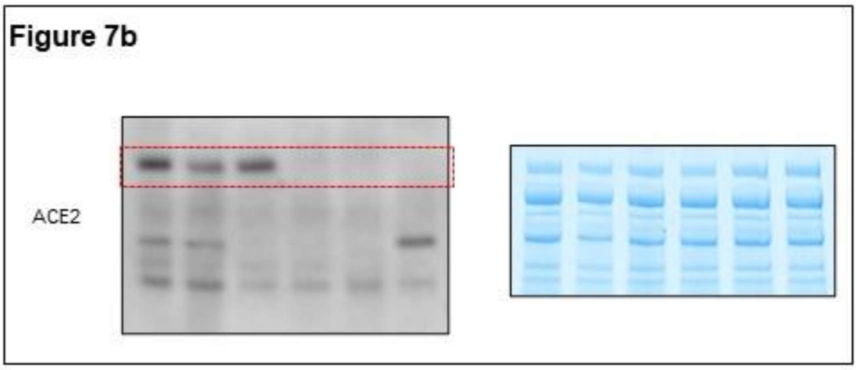

Figure S1

Figure S1a

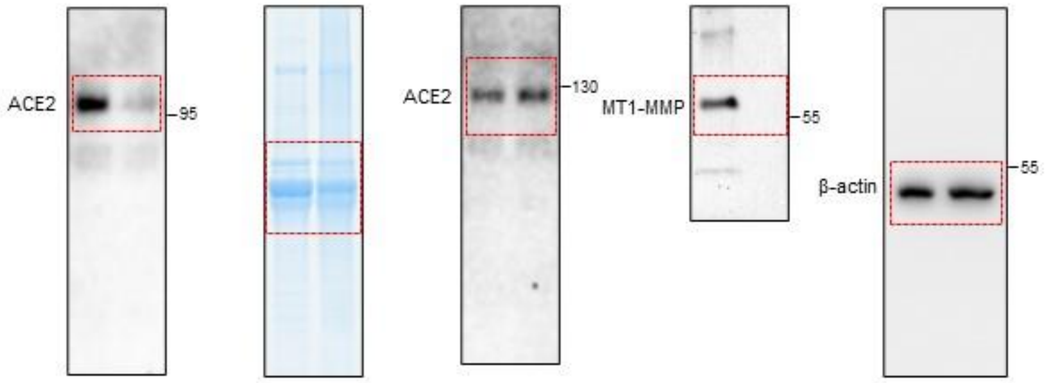

Figure S1b

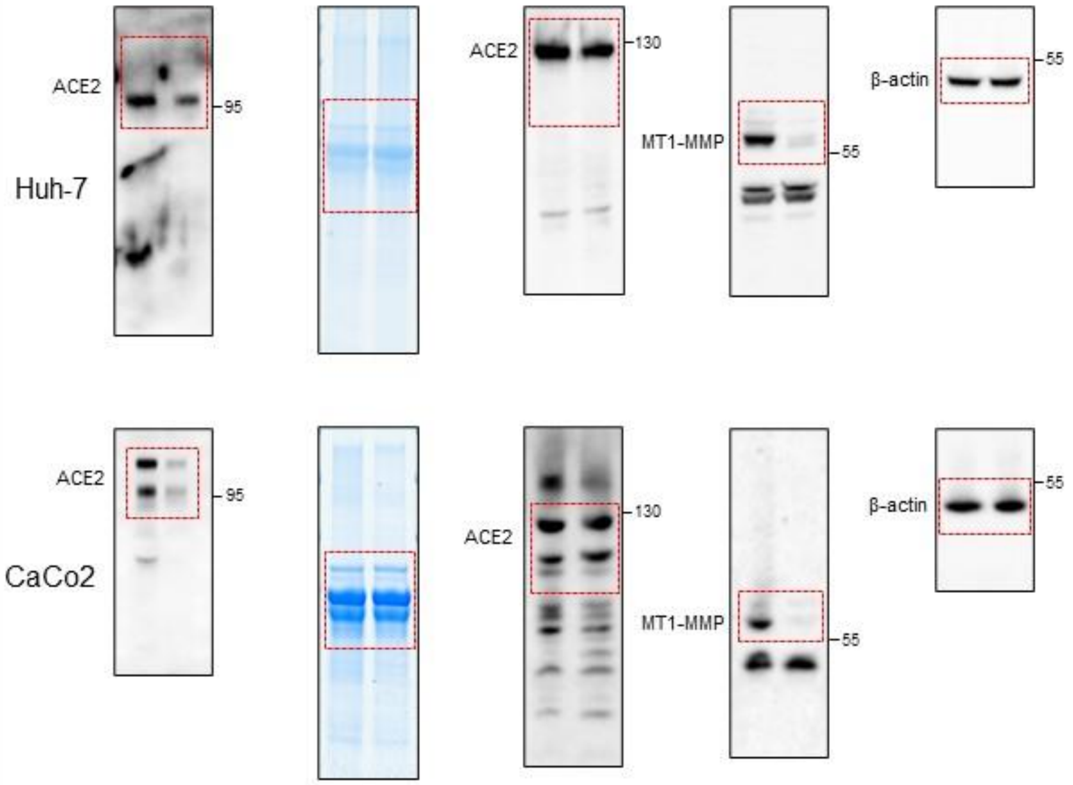

Figure S2

Figure S2a

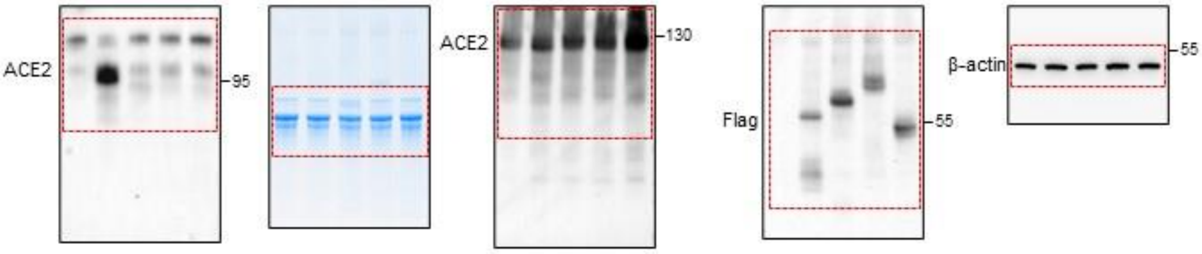

Figure S2b

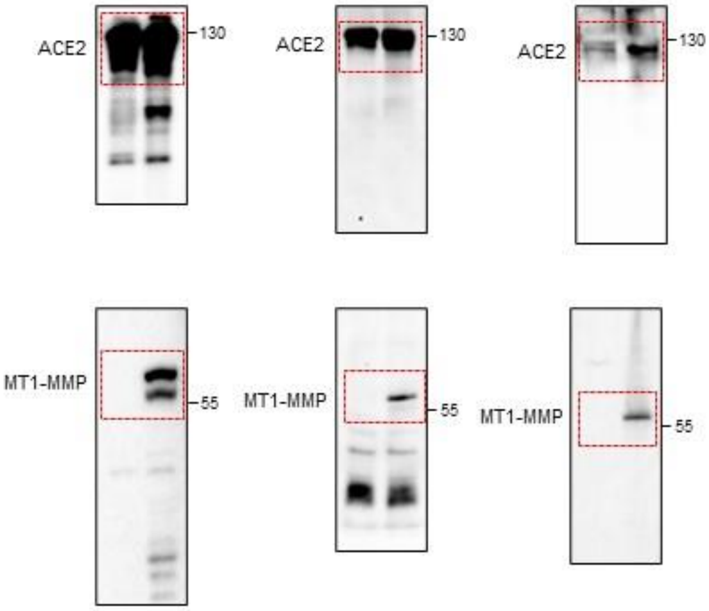

Figure S2c

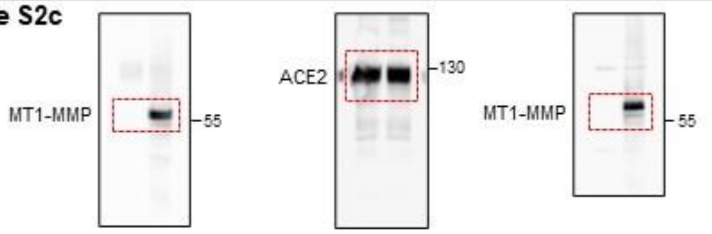

Figure S3

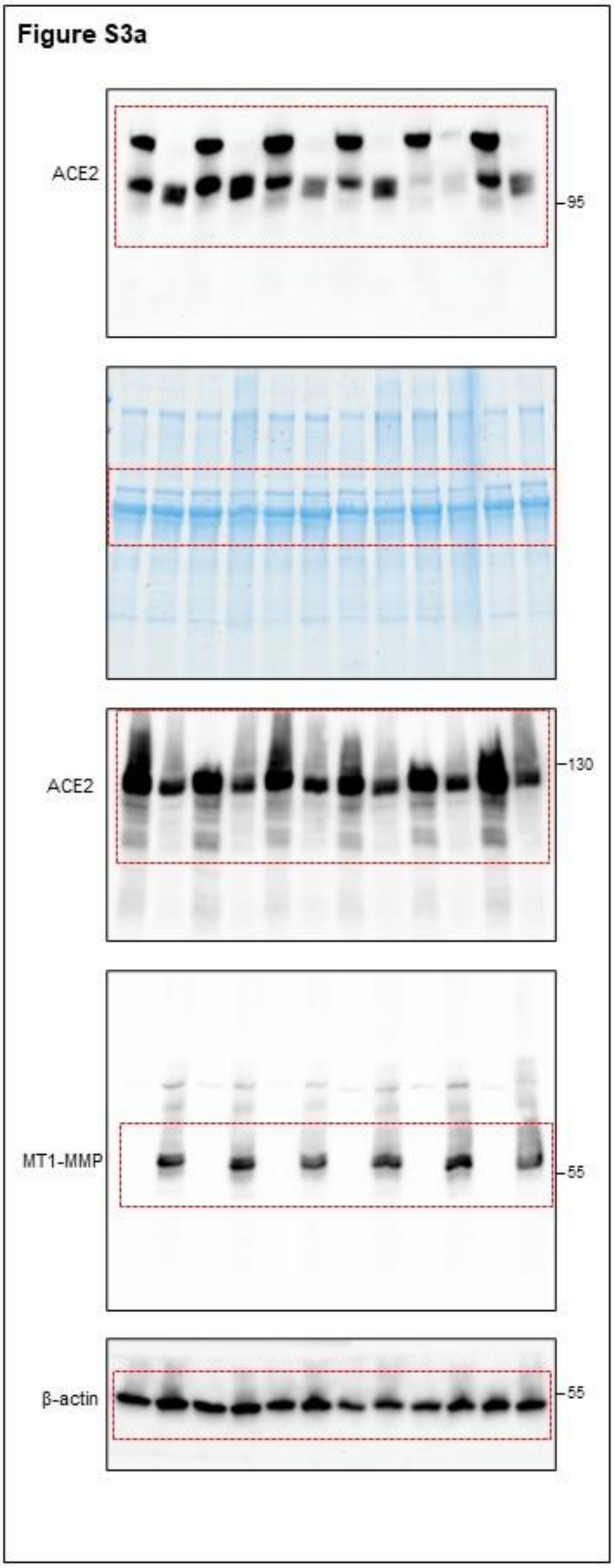
Figure S3a
ACE2
95
ACE2
130
MT1-MMP
55
β-actin
55

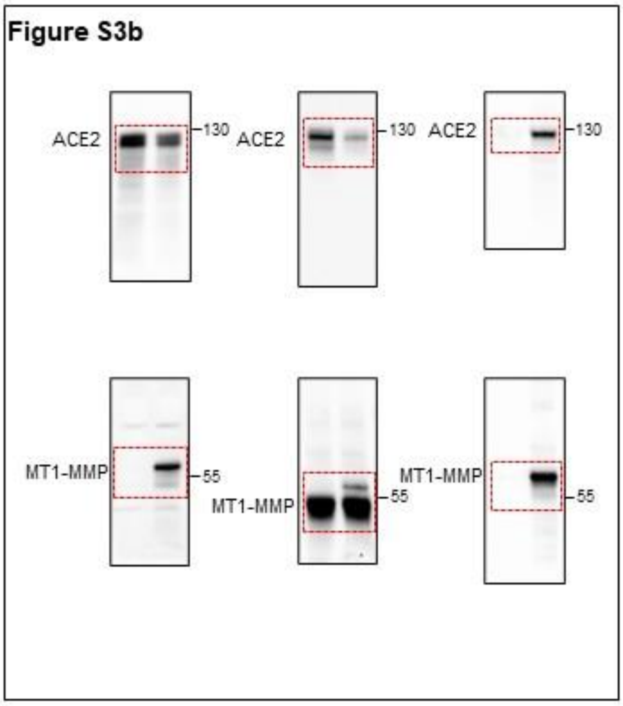
Figure S3b
ACE2
130
ACE2
130
ACE2
130
MT1-MMP
55
MT1-MMP
55
MT1-MMP
55

Figure S6

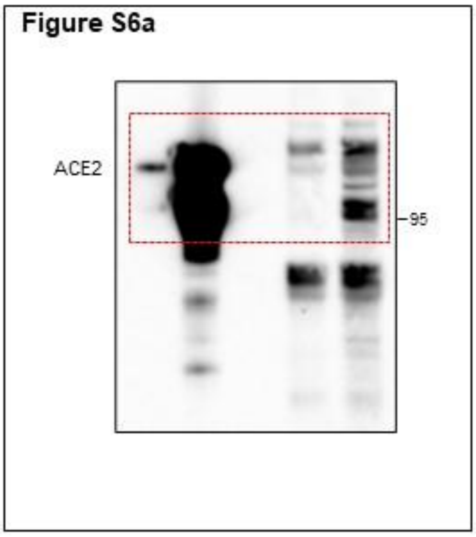

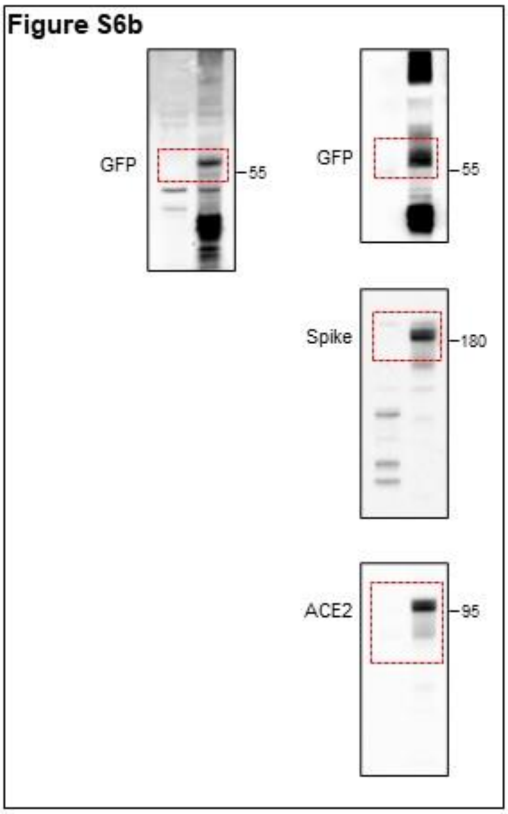

Figure S10

Figure S10a

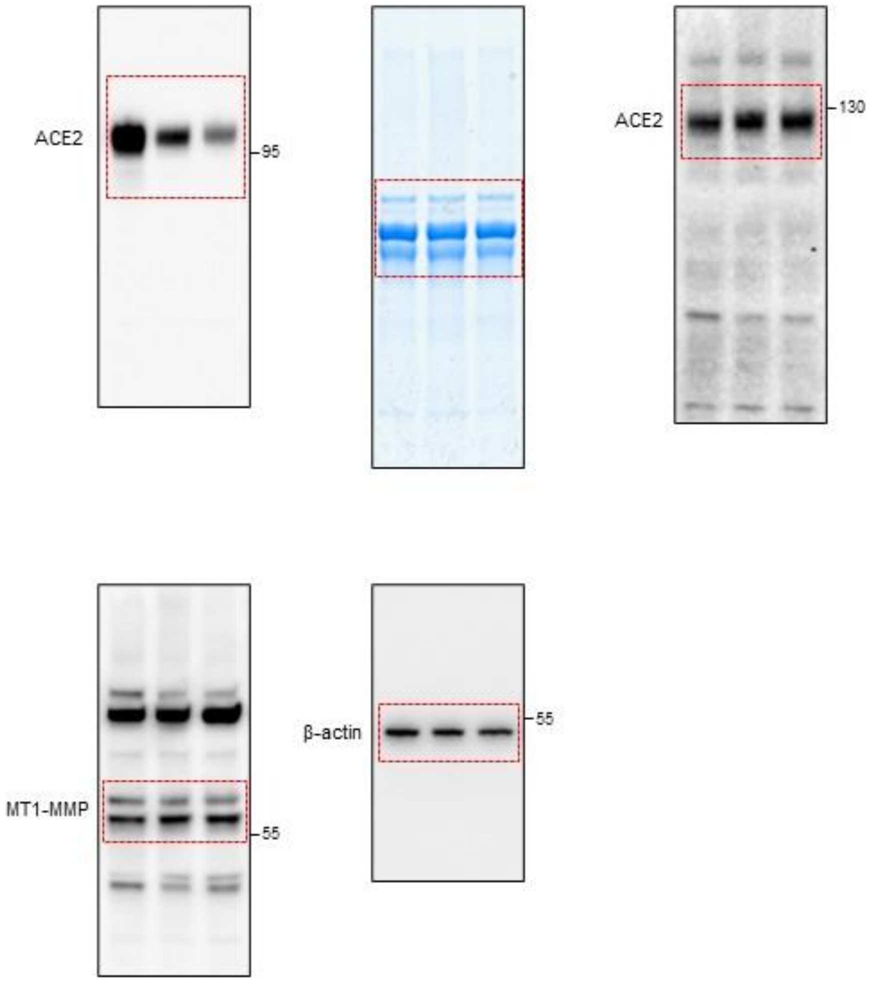

Figure S13

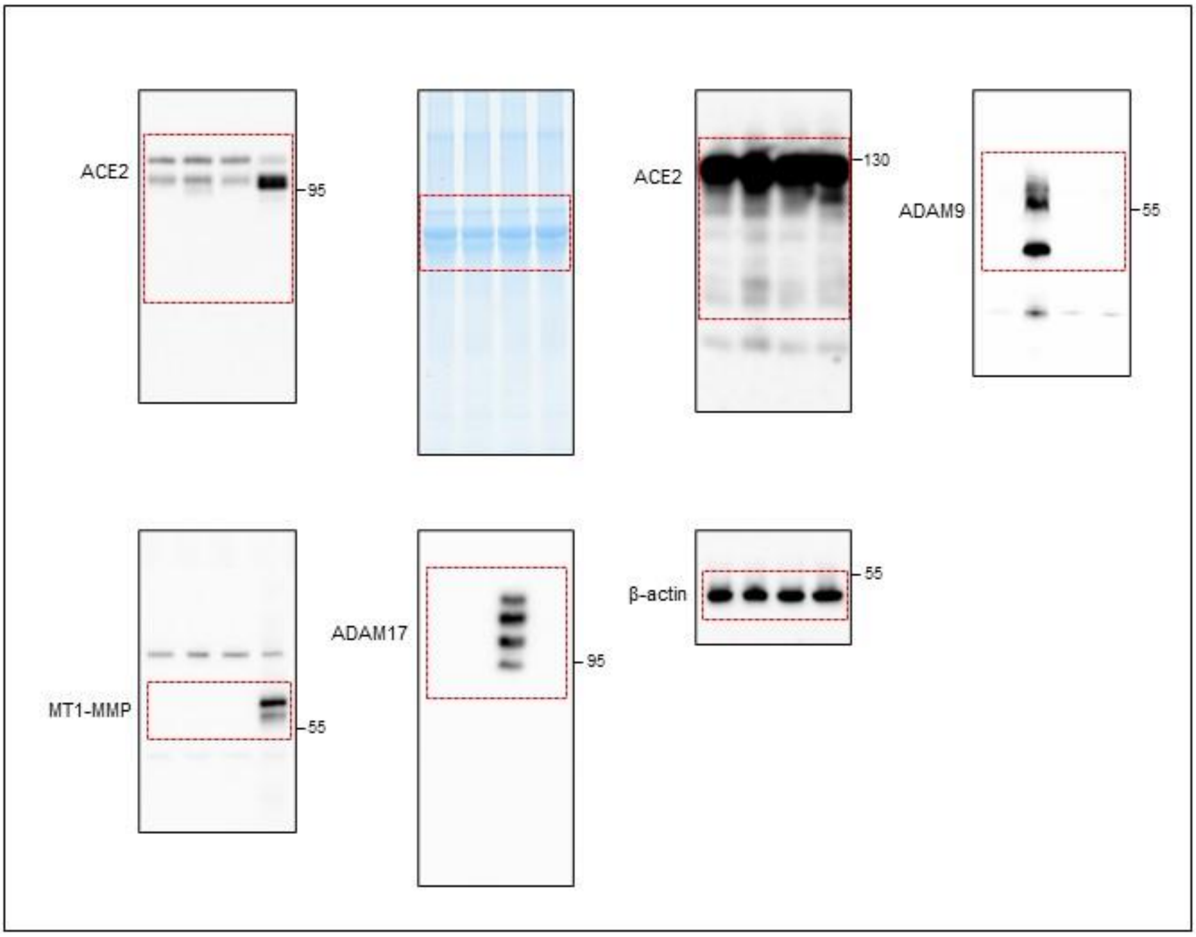

Figure S14

Figure S14a

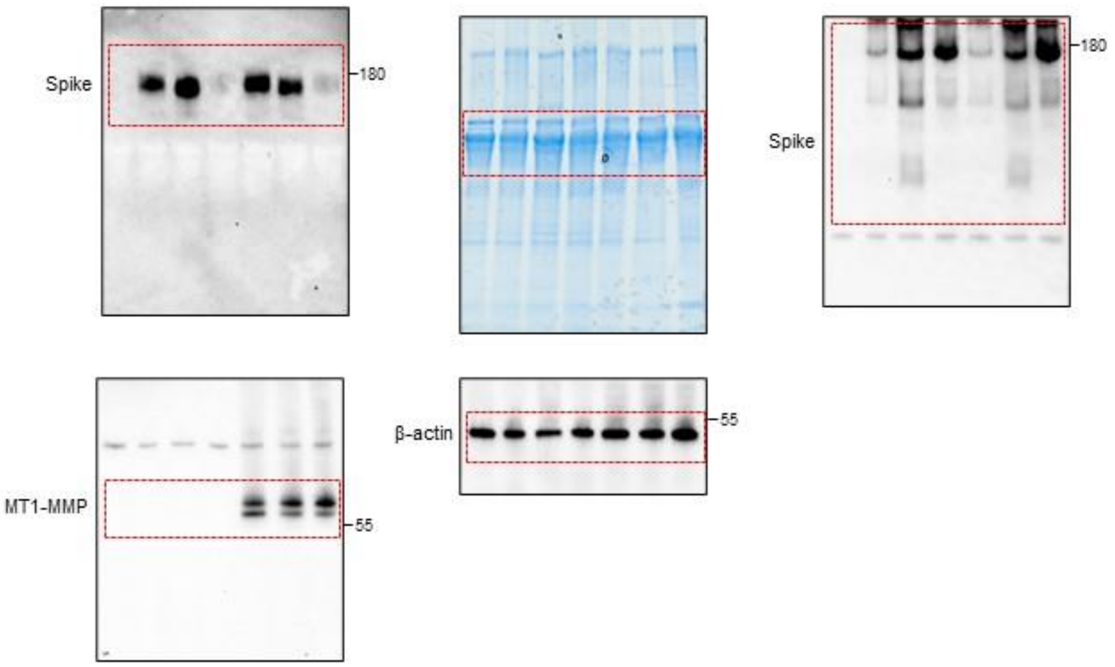

Figure S14b

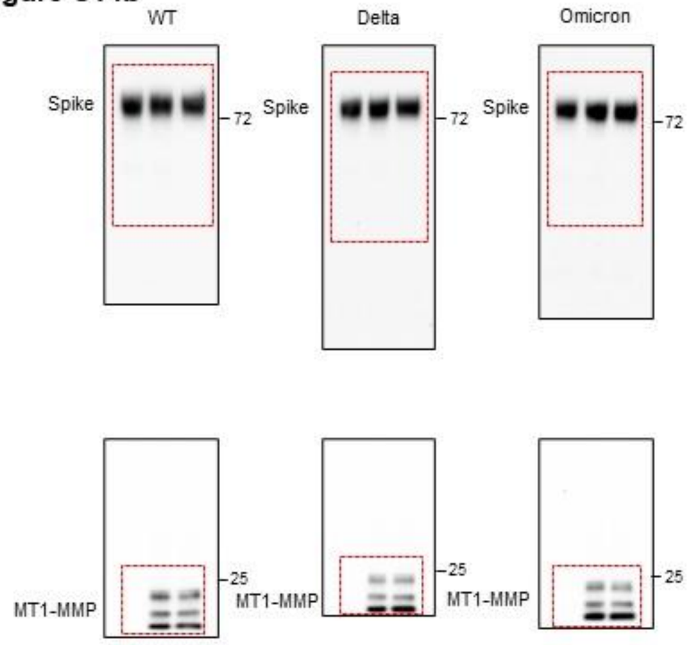

Supplement: Supplementary file 1 — Supplementary Information [file 41467_2022_35590_MOESM1_ESM.pdf]
