# Supplementary material for: Control of SARS-CoV-2 infection by MT1-MMP-mediated shedding of ACE2
Source: Nat Commun. 2022 Dec 23;13:7907. doi: 10.1038/s41467-022-35590-x (PMC9780620; doi:10.1038/s41467-022-35590-x)
Supplement: Supplementary file 3 — Reporting Summary [file 41467_2022_35590_MOESM3_ESM.pdf]

nature portfolio

Corresponding author(s): Wong Hoi Leong Xavier, Yuan Shuofeng,

Last updated by author(s): Dec 6, 2022

Reporting Summary

Nature Portfolio wishes to improve the reproducibility of the work that we publish. This form provides structure for consistency and transparency in reporting. For further information on Nature Portfolio policies, see our Editorial Policies and the Editorial Policy Checklist.

Statistics

For all statistical analyses, confirm that the following items are present in the figure legend, table legend, main text, or Methods section.

n/a	Confirmed	
☐	☒	The exact sample size (*n*) for each experimental group/condition, given as a discrete number and unit of measurement
☐	☒	A statement on whether measurements were taken from distinct samples or whether the same sample was measured repeatedly
☐	☒	The statistical test(s) used AND whether they are one- or two-sided<br>*Only common tests should be described solely by name; describe more complex techniques in the Methods section.*
☒	☐	A description of all covariates tested
☐	☒	A description of any assumptions or corrections, such as tests of normality and adjustment for multiple comparisons
☐	☒	A full description of the statistical parameters including central tendency (e.g. means) or other basic estimates (e.g. regression coefficient) AND variation (e.g. standard deviation) or associated estimates of uncertainty (e.g. confidence intervals)
☐	☒	For null hypothesis testing, the test statistic (e.g. *F*, *t*, *r*) with confidence intervals, effect sizes, degrees of freedom and *P* value noted<br>*Give P values as exact values whenever suitable.*
☒	☐	For Bayesian analysis, information on the choice of priors and Markov chain Monte Carlo settings
☒	☐	For hierarchical and complex designs, identification of the appropriate level for tests and full reporting of outcomes
☐	☒	Estimates of effect sizes (e.g. Cohen's *d*, Pearson's *r*), indicating how they were calculated

Our web collection on statistics for biologists contains articles on many of the points above.

Software and code

Policy information about availability of computer code

Data collection	qPCR analyses were performed by the ABI ViiA 7 real-time PCR system (Applied Biosystems).
Data analysis	GraphPad Prism V8 for Window OS was used for statistical analyses.<br>qPCR results were analyzed by ViiA 7 Real-time PCR system software (QuantStudio Software v1.6.1)<br>Confocal imaging was performed and analyzed by LAS X microscope software (version 3.7.4_23463)<br>Analysis of single cell data:<br>R version 3.6.0<br>Rstudio version 1.1.463 for macOS Code and functions provided by the following R packages: Seurat, dplyr, plyr, stringr, DESeq2, ggplot2, ggbeeswarm, ggpubr.

For manuscripts utilizing custom algorithms or software that are central to the research but not yet described in published literature, software must be made available to editors and reviewers. We strongly encourage code deposition in a community repository (e.g. GitHub). See the Nature Portfolio guidelines for submitting code & software for further information.

Data

Policy information about availability of data

All manuscripts must include a data availability statement. This statement should provide the following information, where applicable:

- Accession codes, unique identifiers, or web links for publicly available datasets
- A description of any restrictions on data availability
- For clinical datasets or third party data, please ensure that the statement adheres to our policy

The raw data that support the findings of this study can be found in the source data provided with this paper. The datasets analysed during the current study are available in FigShare (https://doi.org/10.6084/m9.figshare.12436517, https://doi.org/10.6084/m9.figshare.13200278, and https://doi.org/10.6084/m9.figshare.14938755) and Human Protein Atlas.

Human research participants

Policy information about studies involving human research participants and Sex and Gender in Research.

Reporting on sex and gender	The current study mainly focused on the effect of age on the level of circulating MT1-MMP and soluble ACE2. Considering the lack of evidence for the impact of sex on SARS-CoV-2 infection, we included both female and male participants for the analysis. The information regarding the sex of each participant was collected by self-reporting via a questionnaire.
Population characteristics	21 young (mean = 29.2 years; 13 males & 8 females) and 23 healthy elderly (mean = 80.1 years; 15 males & 8 females), mainly Asian Chinese, were recruited from Guanfu Cancer Hospital in Jinhua, Zhejiang Province, CHINA. They were all healthy, non-obese, with a BMI < 24.
Recruitment	Patients recruited for the normal physical examination, without any patient selection, obtained informed consent from each individual.
Ethics oversight	The study protocol was conducted following the ethical guidelines of the hospital ethics committee, and the Zhejiang Provincial Health Committee approved the procedure. The protocol is in compliance with the Helsinki declaration.

Note that full information on the approval of the study protocol must also be provided in the manuscript.

Field-specific reporting

Please select the one below that is the best fit for your research. If you are not sure, read the appropriate sections before making your selection.

☒ Life sciences ☐ Behavioural & social sciences ☐ Ecological, evolutionary & environmental sciences

For a reference copy of the document with all sections, see nature.com/documents/nr-reporting-summary-flat.pdf

Life sciences study design

All studies must disclose on these points even when the disclosure is negative.

Sample size	We selected the sample size, based on our extensive experience with animal models and endpoints (Guo, X. et al. Regulation of age-associated insulin resistance by MT1-MMP-mediated cleavage of insulin receptor. Nat Commun 13, 3749 (2022)). We make sure that no more animals than necessary were used. Sample size were chosen to generate reproducible results with desirable significance (0.05) and power (>90%).
Data exclusions	No data was excluded from the manuscript.
Replication	All of the experimental results were replicated as indicated in figure legends. For in vitro experiments, each experiment was independently repeated at least three times. Only biological replicates were plotted and used for statistical analyses.
Randomization	Tissues from independently and randomly chosen mice at comparable developmental stages and sexes were collected for analyses and none of the samples was excluded. For the experiments involving transgenic animals, further allocations were based on the genotype of mice.<br>For the human samples, all samples were allocated according to the subtypes (e.g. young vs elderly subjects, infected vs uninfected subjects)
Blinding	For molecular studies including western blotting and qPCR analyses, the experiments were not blinded, due to careful experimental setup and design.<br>During the phenotyping experiments of transgenic mice, the experiments were performed blinded and the genotype was only disclosed after data analyses. All histology scoring was performed by two blinded researchers.

Reporting for specific materials, systems and methods

We require information from authors about some types of materials, experimental systems and methods used in many studies. Here, indicate whether each material, system or method listed is relevant to your study. If you are not sure if a list item applies to your research, read the appropriate section before selecting a response.

Materials & experimental systems

n/a	Involved in the study
☐	☒ Antibodies
☐	☒ Eukaryotic cell lines
☒	☐ Palaeontology and archaeology
☐	☒ Animals and other organisms
☒	☐ Clinical data
☒	☐ Dual use research of concern

Methods

n/a	Involved in the study
☒	☐ ChIP-seq
☒	☐ Flow cytometry
☒	☐ MRI-based neuroimaging

Antibodies

Antibodies used

anti-SARS-CoV-2 spike antibody (40150-R007, Sino Biological, 1:1000); anti-ACE2 antibody (AF933, R&D System, 1:2000); anti-ACE2 antibody (EPR24705-45, ab272500, Abcam,1:500); anti-MMP14 antibody (EP1264Y, ab51074, Abcam, 1:2000); anti-FLAG tag antibody (66008-3-Ig, clone no. 2B3C4, Proteintech, 1:2000) CloneNo.2B3C4; anti-GFP tag antibody (50430-2-AP, Proteintech, 1:2000); β-actin antibody (sc-47778, Santa Cruz, 1:2000); anti-ADAM9 antibody (sc-377233, Santa Cruz, 1:1000); anti-ADAM17 antibody (sc-390859, Santa Cruz, 1:1000); mouse anti-rabbit IgG-HRP antibody (sc-2357, Santa Cruz, 1:5000); mouse anti-goat IgG-HRP antibody (sc-2354, Santa Cruz, 1:5000); m-IgGκ BP-HRP antibody (sc-516102, Santa Cruz, 1:5000); Alexa Fluor 568-conjugated rabbit anti-goat (A11079, Invitrogen, 1:500); Alexa Fluor 647-conjugated donkey anti-rabbit (A31573, Invitrogen, 1:500); Anti-NP antibody was kindly provided by the laboratory of Dr. Shuofeng Yuan (1:5000, Riva, L. et al. Discovery of SARS-CoV-2 antiviral drugs through large-scale compound repurposing. Nature 586, 113–119 (2020).; 3A2 antibody was kindly provided by the laboratory of Dr. Xin Ge

Validation

Commercial available Western blot and immunofluorescence antibodies were selected based on their antigen specificity and suggested application as described on the manufacturer's website and data sheets. Secondary antibodies and others were validated by the manufacturers for the different detection methods. All antibody concentrations for staining were optimized by titrating down each reagent starting at the manufacturer's recommendation. The optimal amounts of the reagents were defined by (i) no minimal to no background signal (western Blot, immunofluorescence).
anti-SARS-CoV-2 spike antibody (Suitable for IF, WB, ELISA; reacts with SARS-CoV-2 Spike S1;https://tw.sinobiological.com/antibodies/cov-spike-40150-r007
anti-ACE2 antibody (Suitable for IHC, IP, WB, Flow; reacts with Human, Mouse, Rat, Hamster; https://www.rndsystems.com/products/human-mouse-rat-hamster-ace-2-antibody_af933
anti-ACE2 antibody (Suitable Flow, IP, WB, IHC-P, ICC/IF; reacts with Human; https://www.abcam.com/ace2-antibody-epr24705-45-ab272500.html
anti-MMP14 antibody (Suitable for IHC, IP, Flow, WB, ICC/IF; reacts with: Mouse, Rat, Human; https://www.abcam.com/mmp14-antibody-ep1264y-ab51074.html
anti-FLAG tag antibody (Suitable for WB, RIP, IP, IHC, IF, CoIP, ELISA; reacts with Human, Mouse; https://www.ptglab.com/products/Flag-tag-Antibody-66008-3-Ig.htm
anti-GFP tag antibody (Suitable for WB, RIP, IP-MS, IP, IHC, IF, CoIP, ChIP, ELISA; reacts with Aequorea Victoria, Recombinant Protein, Mouse; https://www.ptglab.com/products/eGFP-Antibody-50430-2-AP.htm
β-actin antibody (Suitable for WB, IP, IF, IHC; reacts with mouse, rat, human, avian, bovine, canine, porcine, rabbit, Dictyostelium discoideum, Physarum polycephalum; https://www.scbt.com/p/beta-actin-antibody-c4
nti-ADAM9 antibody (Suitable for WB, IP, IF, IHC, ELISA; reacts with mouse, rat, human; https://www.scbt.com/p/adam9-antibody-g-1
anti-ADAM17 antibody (Suitable for WB, IP, IF, IHC, ELISA; reacts with human; https://www.scbt.com/p/tace-antibody-b-6
The Anti-NP antibody was validated in the following articles: Riva, L. et al. Discovery of SARS-CoV-2 antiviral drugs through large-scale compound repurposing. Nature 586, 113–119 (2020)
The 3A2 antibody was validated in the following articles: Chow, C. F. W. et al. Body weight regulation via MT1-MMP-mediated cleavage of GFRAL. Nat Metab 1–10 (2022) doi:10.1038/s42255-022-00529-5.

Eukaryotic cell lines

Policy information about cell lines and Sex and Gender in Research

Cell line source(s)

Human embryonic kidney (HEK)293T, Caco-2, HK-2 cells, A549 and Huh-7 cells were purchased from the American Type Culture Collection (ATCC, USA; CRL-3216 for HEK293T; CRL-8024 for Huh7; HTB-37 for Caco2, CCL158 for A529). Human bronchial epithelial cells were purchased from ScienCell (cat no. 3210); the sex origins of cell lines are unknown.

Authentication

all ATCC cell lines undergo authentication tests during the accessioning process (STR profiling). This process is described in the online ATCC brochure "Maintaining High Standards in Cell Culture". Human Bronchial Epithelial Cells from ScienCell were

	not authenticated.
Mycoplasma contamination	The cells were tested negative for mycoplasma contamination
Commonly misidentified lines (See ICLAC register)	No commonly misidentified cell lines were used

Animals and other research organisms

Policy information about studies involving animals; ARRIVE guidelines recommended for reporting animal research, and Sex and Gender in Research

Laboratory animals	Wild-type mice on C57BL/6J background were from the Laboratory Animal Services Centre of The Chinese University of Hong Kong. Mmp14-/- mice in C57BL6 background were kindly provided by Prof. Zhou Zhongjun. All animals and their borne pups were housed in the animal house at Hong Kong Baptist University and maintained on a 12-h (h) light/dark cycle with constant ambient temperature (22 –24 °C) and humidity (~60%). They were fed with standard laboratory chow, and applied with water ad libitum. All animals were between 6 and 12 weeks old unless otherwise stated in the age group experiments.
Wild animals	No wild animals was used.
Reporting on sex	Mice of both sexes were used in the experiments.
Field-collected samples	This study did not involve samples collected from the fields.
Ethics oversight	All animal experiments were performed according to the guideline of the Committee on the Use of Human & Animal Subjects in Teaching & Research at Hong Kong Baptist University and procedures were approved by the Department of Health under Hong Kong legislation.

Note that full information on the approval of the study protocol must also be provided in the manuscript.
